# The Combined Analysis of Transcriptome and Antioxidant Enzymes Revealed the Mechanism of EBL and ZnO NPs Enhancing *Styrax tonkinensis* Seed Abiotic Stress Resistance

**DOI:** 10.3390/genes13112170

**Published:** 2022-11-20

**Authors:** Ze-Mao Liu, Mohammad Faizan, Chen Chen, Li-Hong Zheng, Fang-Yuan Yu

**Affiliations:** 1 Collaborative Innovation Centre of Sustainable Forestry in Southern China, College of Forest Science, Nanjing Forestry University, Nanjing 210000, China; 727046890@njfu.edu.cn (Z.-M.L.); zhenglh@njfu.edu.cn (L.-H.Z.); 2 Botany Section, School of Sciences, Maulana Azad National Urdu University, Hyderabad 500032, India; mohammadfaizan@manuu.edu.in; 3 School of Landscape and Horticulture, Yangzhou Polytechnic College, Yangzhou 225009, China; cc0212@njfu.edu.cn

**Keywords:** antioxidant enzyme activity, EBL, LEA, *Styrax tonkinensis*, ZnO NPs

## Abstract

As global climate change worsens, trees will have difficulties adapting to abiotic pressures, particularly in the field, where environmental characteristics are difficult to control. A prospective commercial and ornamental tree species, *Styrax tonkinensis*, has its seed oil output and quality reduced as a result, which lowers the economic benefits. This necessitates growers to implement efficient strategies to increase the seeds of woody biofuel species’ tolerance to abiotic stress. Numerous studies have shown that ZnO nanoparticles (NPs), a new material, and BRs assist plants to increase their resilience to abiotic stress and subsequently adapt to it. However, there have not been many investigations into *S. tonkinensis* seed resistance. In this study, we examined the changes in antioxidant enzyme activities and transcriptomic results of *S. tonkinensis* seeds throughout the seed development period to investigate the effects of 24-epibrassinolide (EBL), one of the BRs, and ZnO NPs treatments alone or together on the stress resistance of *S. tonkinensis* seeds. On 70, 100, and 130 days after flowering (DAF), spraying EBL or ZnO NPs increased the activity of antioxidant enzymes (POD, SOD, and CAT) in *S. tonkinensis* seeds. Moreover, when the EBL and ZnO NPs were sprayed together, the activities of antioxidant enzymes were the strongest, which suggests that the positive effects of the two can be superimposed. On 70 and 100 DAF, the EBL and ZnO NPs treatments improved seed stress resistance, mostly through complex plant hormone crosstalk signaling, which includes IAA, JA, BR, and ABA signaling. Additionally, ABA played an essential role in hormone crosstalk, while, on 130 DAF, due to the physiological characteristics of seeds themselves in the late stage of maturity, the improvement in seed stress resistance by EBL and ZnO NPs was related to protein synthesis, especially late embryogenesis-abundant protein (LEA), and other nutrient storage in seeds. Spraying EBL and ZnO NPs during the seed growth of *S. tonkinensis* could significantly increase seed stress resistance. Our findings provide fresh perspectives on how cultural practices can increase abiotic stress tolerance in woody seedlings.

## 1. Introduction

In 1979, a set of plant steroid hormones known as brassinosteroids (BRs) were originally discovered and extracted from the pollen of the *Brassica napus* plant [1]. As the sixth class of plant hormones, BRs have been recognized as a key player in regulating plant growth and development, including seed germination, architecture, senescence, flowering time, seed yield, cell cycle progression, cell elongation, and tolerance to various abiotic and biotic stresses, etc. [2,3]. Among them, improvement in plant stress resistance to abiotic stress is of particular interest. Studies have shown that BRs can enhance plant tolerance to a variety of abiotic stimuli, such as heavy metals, cold, drought, salt, and other stresses, by boosting production of osmotic regulating chemicals, enhancing photosynthesis, and fine-tuning stress-related transcriptional networks [4,5,6,7].

Nanoparticles (NPs), which range in size from 1 to 100 nanometers, have unique physicochemical characteristics that enable them to enter plants through the aqueous sheath and stomata of leaves and then decompose into their ionic state for transfer through the xylem to the rest of the plant [8]. Therefore, NPs’ potential to be exploited to affect plant growth and development is thus not a difficult concept to grasp [9]. One of the most popular nanoparticles is ZnO NPs, and, in a short amount of time, production has expanded from 550 tons to 33,400 tons and is still rising [10]. There are currently a growing number of papers indicating that ZnO NPs can be employed to alleviate abiotic stress in plants [11,12,13,14,15]. Additionally, to help plants resist abiotic challenges, ZnO NPs enhance synthesis of photosynthetic pigments, control redox status, strengthen antioxidant defense mechanisms, and regulate primary glucose metabolism and phytohormone signaling [16,17].

*S. tonkinensis*, a member of the Styracaceae family of deciduous trees, is primarily found in Southeast Asia, including Vietnam and China [18]. The seeds of *S. tonkinensis* have high oil content, and their seed oil meets US, German, EU, and Chinese biodiesel standards [19,20]. The resin of *S. tonkinensis* is used as perfume and medicine and has high potential economic value [21,22]. Additionally, *S. tonkinensis*’s lovely white blossoms make it suitable for use as an ornamental plant. As a result, *S. tonkinensis* is a tree species with high potential for commercial and ornamental value. Its strong potential as a species for woody biodiesel with high oil content is particularly interesting [23,24].

Since they are immobile, plants are frequently challenged by a variety of abiotic stresses, which have a detrimental effect on their survival, growth, and reproduction [25]. Additionally, the detrimental effects of abiotic pressures are exacerbated by climate change and environmental contamination [26,27]. As a result, *S. tonkinensis* and other woody biodiesel species’ oil production and quality would decline, diminishing the economic advantages. The different abiotic stressors that the seeds may experience during development are, therefore, one of the key factors impacting the seed quality and production of *S. tonkinensis*, especially in the field setting where fine management is challenging. Therefore, it is essential to investigate easy-to-implement culture methods for dealing with potential abiotic stress by enhancing seed resilience throughout development. Transcriptome and oxidase activity measurements were used to investigate the impact of spraying zinc oxide NPs and EBL on the ability of seeds of *S. tonkinensis* to withstand abiotic stress during seed development in this work. Furthermore, there has been a great deal of research conducted on ZnO NPs in crops but very little on tree species. Meanwhile, fewer studies have been conducted to investigate the effects of ZnO NPs on plant growth and development through a transcriptomic method. This study will also provide insights into possible application of ZnO NPs in woody species through a combined transcriptomic and physiological indicator approach.

## 2. Material and Methods

### 2.1. Site Information and Experimental Design

The experiments were conducted in the Styracaceae Germplasm Repository (32°54′ N, 118°50′ E) situated in Nanjing, China. The area is characterized by a humid north subtropical monsoon climate, with average annual temperatures of 15.3 °C and 970 mm of annual rainfall. Summertime highs range from 36 to 38 °C, while wintertime lows range from −8 to −10 °C. Hilly terrain characterizes the experimental site, and the soil fertility is favorable for plant development [23].

ZnO NPs were procured from Shanghai Yi En Chemical Technology Co., Ltd. (Shanghai, China). The specific configuration methods of EBL and ZnO NPs solutions refer to the previous practice of our research group [23,28]. Moreover, the concentrations of EBL and ZnO NPs that may increase the stress resistance of plant seeds were chosen based on the findings of our prior studies [23,28]. Then, 6-year-old *S. tonkinensis* plants that bear fruits were chosen as the research materials. The experiment was performed using a two-factorial (EBL and ZnO NPs) randomized block design with three replicates for each treatment. A total of four treatments were set (Table 1). On the 65th day after anthesis (DAF) (25 July 2019), the 95 DAF and the 125 DAF different concentrations of EBL and ZnO NPs were sprayed on the foliage of the sampling trees. Control plants were treated with distilled water only. After 5 days of treatment, several fruits were randomly taken from all directions on the sampling trees, and the seeds were quickly peeled from the fruits. The removed seeds were snap-frozen in liquid nitrogen and then stored in a −80 °C refrigerator. Three biological replicates were performed for each treatment, so a total of 36 samples were obtained. For convenience, the letters represent the different treatments in each period (Supplementary Table S1).

### 2.2. Determination of Physiological Indexes

The soluble protein content was determined by Coomassie brilliant blue B-250 method [29]. The activity of peroxidase (POD), superoxide dismutase (SOD), and catalase (CAT) was determined by the guaiacol colorimetric method, nitroblue tetrazolium (NBT) method, ultraviolet absorption method, respectively [30]. 

### 2.3. RNA Extraction 

Following the manufacturer’s instructions (Invitrogen), total RNA was isolated from the tissue using TRIzol^®^ Reagent (Plant RNA Purification Reagent for plant tissue) and genomic DNA was removed using DNase I (Takara). On 1% agarose gels, RNA deterioration and contamination were observed. Then, using the ND-2000 (NanoDrop Technologies), the 2100 Bioanalyser (Agilent Technologies) evaluated the quality of the total RNA and quantified its integrity and purity. The sequencing library was built exclusively from high-quality RNA samples (OD260/280 = 1.8–2.2, OD260/230 ≥ 2.0, RIN ≥ 8.0, 28S:18S ≥ 1.0, >1 μg).

### 2.4. Library Preparation and Sequencing

Following the manufacturer’s instructions (Illumina, San Diego, CA, USA), RNA purification, reverse transcription, library creation, and sequencing were carried out at Shanghai Majorbio Bio-pharm Biotechnology Co., Ltd. (Shanghai, China). Using 1 g of total RNA, the transcriptome library was created using the TruSeqTM RNA sample preparation Kit from Illumina (San Diego, CA, USA). Shortly, oligo (dT) beads were used to isolate messenger RNA using the poly-A selection method, and, after that, fragmentation buffer was used to complete the process. Second, double-stranded DNA was created using a SuperScript double-stranded cDNA synthesis kit (Invitrogen, CA, USA) with random hexamer primers (Illumina). Consequently, in accordance with Illumina’s library construction protocol, the synthesized cDNA was subjected to end-repair, phosphorylation, and ‘A’ base addition. Libraries were size-selected for cDNA target fragments of 300 bp on 2% Low Range Ultra Agarose, then PCR-amplified for 15 PCR cycles using Phusion DNA polymerase (NEB). After being quantified by TBS380, the paired-end RNA-seq sequencing library was sequenced with the Illumina NovaSeq 6000 sequencer (2 × 150 bp read length).

### 2.5. De Novo Assembly and Annotation

Using the default parameters, fastp (https://github.com/OpenGene/fastp (accessed on 25 December 2019)) was used to trim and quality-control the raw paired-end reads [31]. Then, using Trinity (http://trinityrnaseq.sourceforge.net/ (accessed on 25 December 2019)), de novo assembly was performed using clean data from the samples [32]. After that, BUSCO (Benchmarking Universal Single-Copy Orthologs, http://busco.ezlab.org (accessed on 25 December 2019)) [33], TransRate (http://hibberdlab.com/transrate/ (accessed on 25 December 2019)) [34], and CD-HIT (http://weizhongli-lab.org/cd-hit/ (accessed on 25 December 2019)) [35] were used to evaluate and optimize the assembled transcripts. The procedures for GO annotation are as follows: transcripts to be annotated were searched against NCBI protein non-redundant (NR, ftp://ftp.ncbi.nlm.nih.gov/blast/db/ (accessed on 25 December 2019)), Swiss-ProtGO (http://web.expasy.org/docs/swiss-prot_guideline.html (accessed on 25 December 2019)), Pfam (http://pfam.xfam.org/ (accessed on 25 December 2019)), and GO (http://www.geneontology.org (accessed on 25 December 2019)) databases using BLASTX to identify the proteins that had the highest sequence similarity with the given transcripts to retrieve their function annotations, and typical cut-off E-values less than 1.0 × 10^−5^ were set.

### 2.6. Differential Expression Analysis and Functional Enrichment

The transcripts per million reads (TPM) approach was used to determine the expression level of each gene in order to discover DEGs (differential expression genes) across two different samples/groups. To measure gene abundances, RSEM (http://deweylab.biostat.wisc.edu/rsem/ (accessed on 26 December 2019)) was utilized [36]. DESeq2 was used to perform differential expression analysis, and DEGs with |log2(foldchange)| ≥ 1 and P-adjust < 0.05 were regarded as significantly differentially expressed genes. Additionally, KEGG pathway analysis was performed by Goatools (https://github.com/tanghaibao/Goatools (accessed on 26 December 2019)) and KOBAS (http://kobas.cbi.pku.edu.cn/home.do (accessed on 26 December 2019)) [37]. Fisher exact test was used. A significant KEGG pathway enrichment in the gene set was defined as the corrected *p* value (FDR) < 0.05.

### 2.7. WGCNA Analysis

WGCNA analysis was performed online using the Majorbio Cloud with default parameters [38]. 

### 2.8. Statistics Analysis 

All measurements were set up with three replicates, and their results were shown as mean ± standard deviation. The data processing was completed with Excel 2010. Using SPSS 26.0, one-way analysis of variance (ANOVA) and Duncan’s multiple comparisons were carried out, and significant differences among various treatment groups are denoted by different letters (*p* < 0.05).

## 3. Results

### 3.1. Response of the Antioxidant Enzyme to Treatments

**Figure 1:**
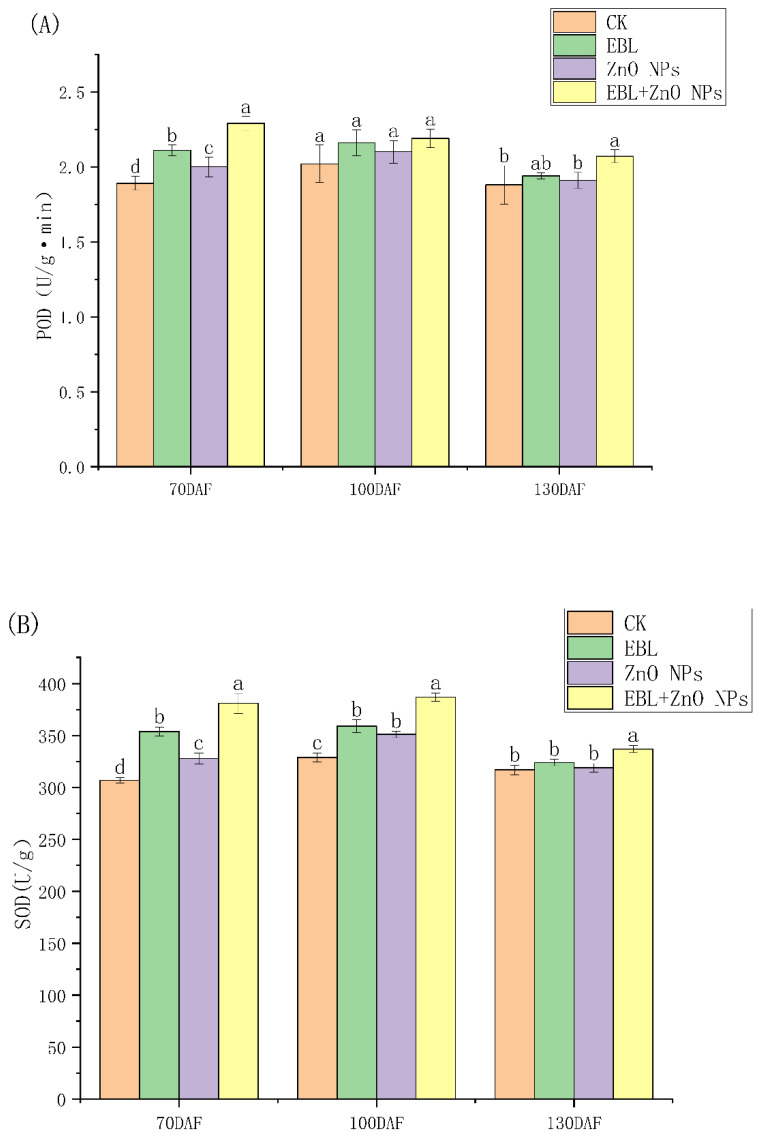

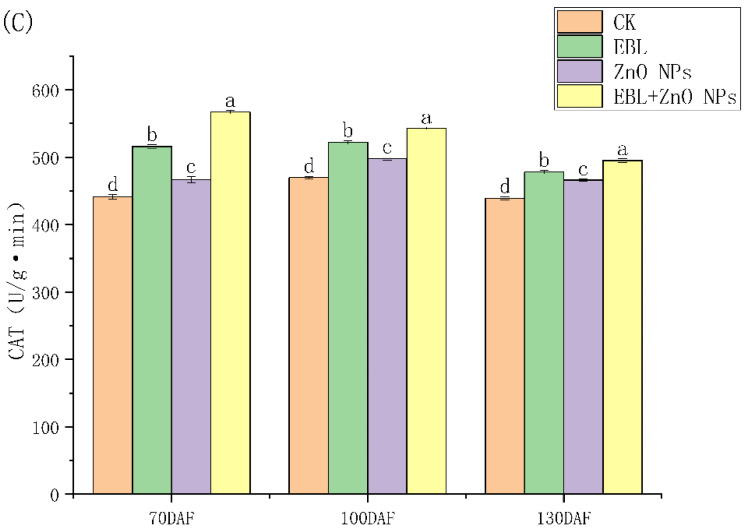
Effects of EBL and ZnO NPs on the antioxidant enzyme activities of *S. tonkinensis* seeds. The antioxidant enzymes are: (**A**): POD; (**B**): SOD and (**C**): CAT, respectively. Note: significant variations between treatments at a given period are shown by different letters. Same below.

Figure 1 shows that EBL and ZnO NPs alone and together almost significantly increased the antioxidant enzyme activities of *S. tonkinensis* seeds in all periods. What is more, when the EBL and ZnO NPs were sprayed together, the activities of antioxidant enzymes were strongest. 

### 3.2. Sequencing, Assembly, and Sequence Analysis

Transcriptome analysis of 36 samples was completed, and a total of 257.34 GB of clean data were obtained. The clean data of each sample were more than 6.28 GB, and the percentage of Q30 base was more than 94.57%. Trinity was used to assemble all the samples of clean data from scratch, optimize, and evaluate the assembly results. The results showed that the number of unigenes obtained by the assembly was 213,566, and the number of transcripts was 329,559 (Table 2). Among all the unigenes, 1786 transcription factors were identified.

**Figure 2:**
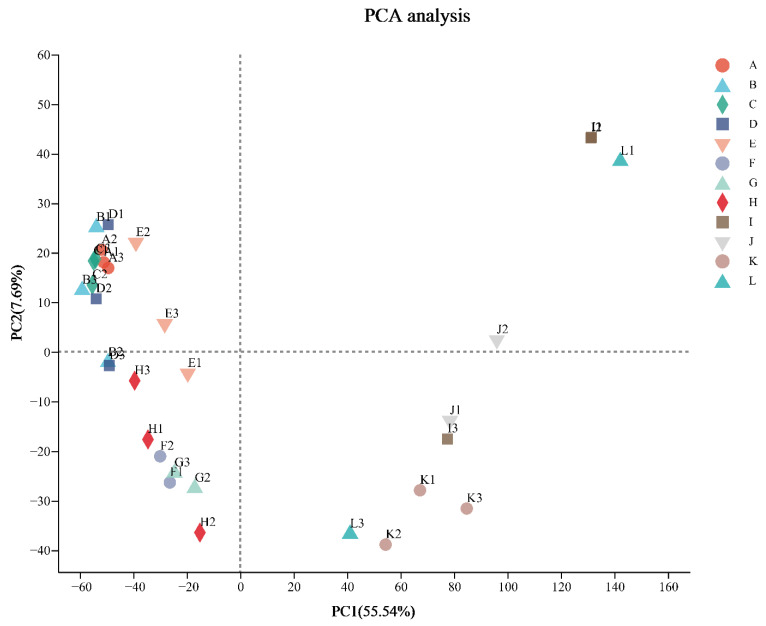
Principal component analysis of all samples.

Almost all three biological replicates had high Pearson’s correlation coefficients (R^2^ = 0.56–0.99) (Supplementary Table S2). Some abnormal replicates (F3, G1, J3, and L2) need to be eliminated for subsequent analysis. Next, principal component analysis was performed on all the remaining samples. As shown in Figure 2, the first principal component accounted for 55.54% of the total variance and clearly separated the third period from the rest. Meanwhile, the second principal component separated the other two periods and accounted for 7.69% of the total variance. Additionally, the outcomes of the four treatments in the first period were more closely clustered in the figure compared to the subsequent two periods.

### 3.3. Differently Expressed Genes Obtained in Different Treatments

**Figure 3:**
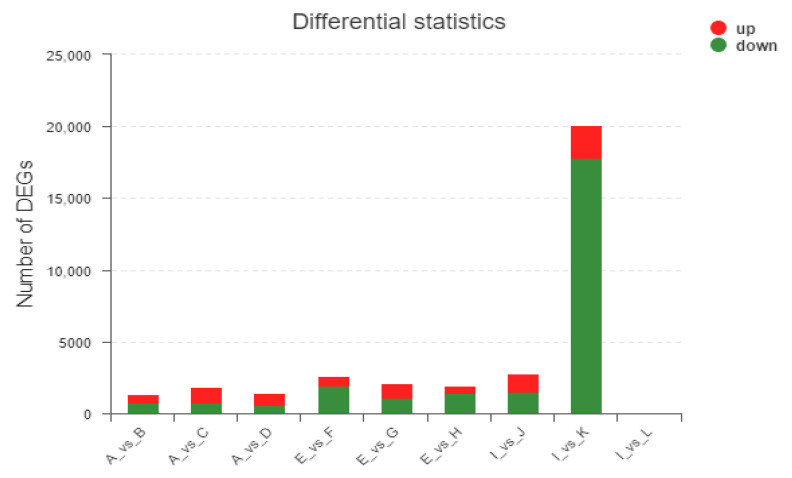
The number of differentially induced genes treated with EBL and ZnO NPs alone or together. Note: the abscissa represents different differential comparison groups, and the ordinate represents the corresponding number of upregulated genes/transcripts. Red means up, and green means down.

Through comparative analysis, DEGs were obtained under different treatments at three growth stages. A total of 231 (155 upregulated and 76 downregulated) and 834 (618 upregulated and 216 downregulated) DEGs were identified at the three growth stages, respectively (Figure 3). It must be noted that the number of genes that differ between I and L is so small that it is hardly visible in the figure.

### 3.4. WGCNA Analysis

**Figure 4:**
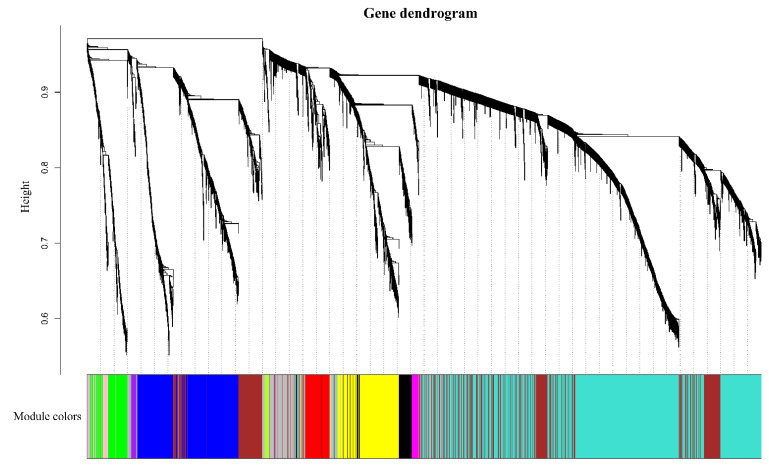
Identification of gene co-expression modules. Note: according to the expression trend of gene/transcripts, gene/transcripts are divided into modules, where a branch represents a gene/transcript and a color represents a module. If the color is gray, it represents genes/transcripts that are not divided into specific modules.

**Figure 5:**
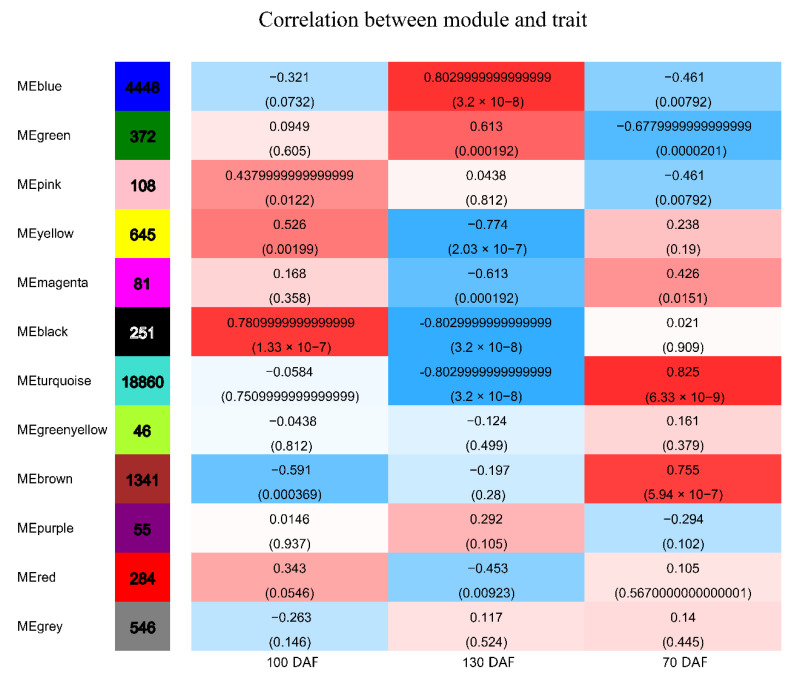
Heat map of the correlation between modules and periods. Note: the number of genes/transcripts in the left column of the figure represents the number of genes/transcripts in the module, and each group of data on the right represents the correlation coefficient and significance P-value between the module and the phenotype (in parentheses).

**Figure 6:**
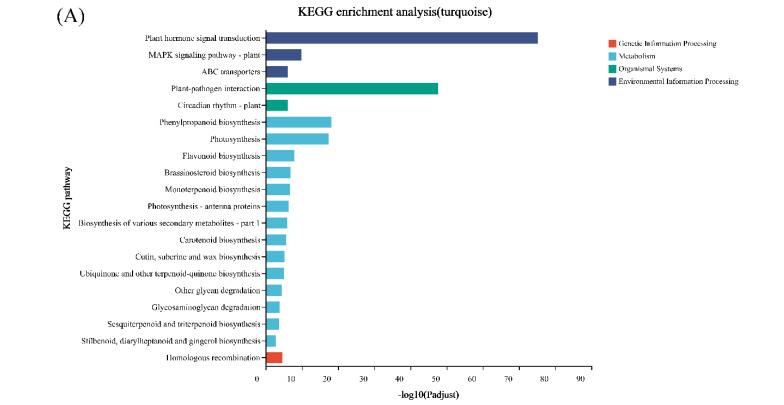

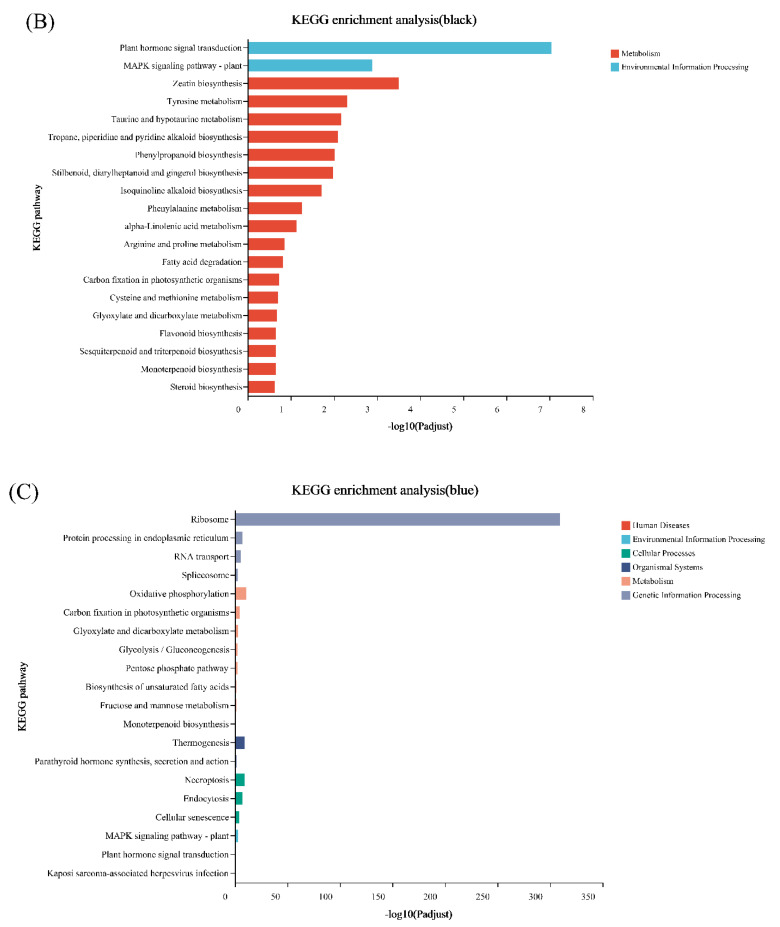
KEGG enrichment analysis of WGCNA modules with the highest correlation with the three stages of seed development of *S. tonkinensis*. The WGCNA module corresponded to the seed development stage as follows: (**A**): MEturquoise corresponded to 70DAF; (**B**): MEblack corresponds to 100DAF and (**C**): MEblue to 130DAF. Note: different colors indicate different branches of the KEGG metabolic pathway, which are metabolism (M), genetic information processing (GIP), environmental information processing (EIP), and biological systems (OS), the same below.

Genes with similar expression patterns were grouped into one module, and a total of 12 modules were identified. The number of genes included in these modules ranged from 46 to 18,860 (Figure 4). Furthermore, the correlation between modules and periods was analyzed (Figure 5). Obviously, the modules with the highest correlation in the three periods (70, 100, and 130 DAF) are MEturquoise, MEblack, and MEblue, in order. Therefore, a module can be used to represent its corresponding period. Then, the three modules were enriched by KEGG (Figure 6). As shown in Figure 5, the enrichment degree of the module for metabolism increased first and then decreased sharply in chronological order. It is worth noting that plant hormone signal transduction is relatively prominent in the first two modules for stress resistance, while the ribosome pathway is prominent in the last module. This shows that, during the first two phases of seed maturation, stress resistance activity in seeds may be more concentrated in hormone signaling, while it is more concentrated in protein synthesis and accumulation during the later stages of seed maturation. 

### 3.5. Search for Key Genes in Plant Hormone Signaling Pathway

**Figure 7:**
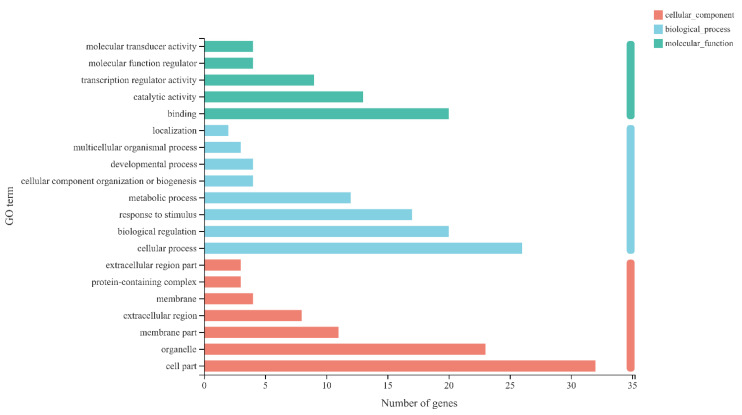
GO annotation of differentially expressed genes involved in plant hormone signal transduction. Note: the ordinate represents the GO term, and the abscissa represents the significance level of enrichment corresponding to the height of the column. The three colors represent three major categories, namely biological process (BP), cellular component (CC), and molecular function (MF).

By using KEGG enrichment to evaluate the differential genes of A VS B, A VS C, A VS D, E VS F, E VS G, and E VS H, 64 genes enriched in plant hormone signal transduction were retrieved (Supplementary Table S3). Then, these 64 genes were subjected to GO annotation analysis. As shown in Figure 7, in CC, most enriched pathways were found in binding (20 genes), catalytic activity (13 genes), and transcription regulator activity (9 genes); in BP, most enriched pathways were found in cellular process (26 genes), biological regulation (20 genes), and response to stimulus (17 genes); in MF, most enriched pathways were found in cell part (32 genes), organelle (23 genes), and membrane part (11 genes).

### 3.6. About the Later Stages of Seed Development

**Figure 8:**
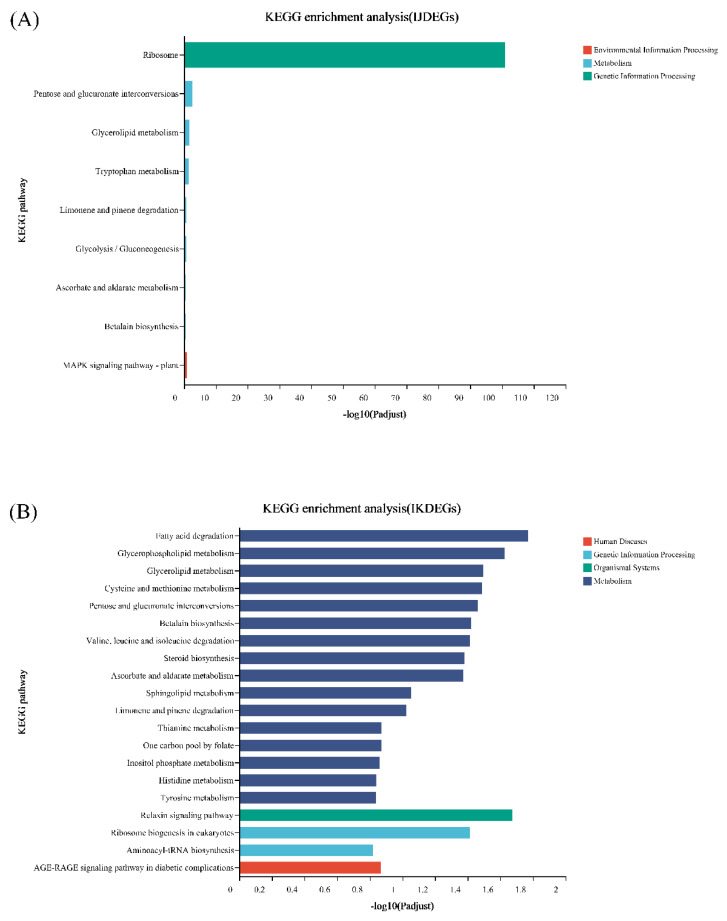

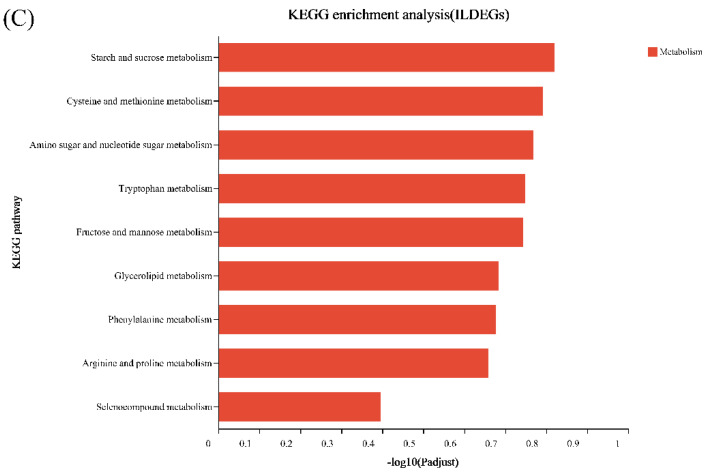
KEGG enrichment of differentially expressed genes between treatment and control in *S. tonkinensis* seeds at the late development stage. (**A**): differentially expressed genes between EBL treatment and control; (**B**): differentially expressed genes between ZnO NPs treatment and control; (**C**): differentially genes between EBL plus ZnO NPs together treatment and control, respectively.

As shown in Figure 8, the KEGG enrichment results of the differential genes of I and J are significantly different from the other two. The differential genes of I VS J and I VS K are enriched in the ribosomal-related pathway, while the differential genes of I and L are all enriched in pathways related to metabolism.

## 4. Discussion

### 4.1. Changes in Antioxidant Enzyme Activities under Different Treatments

Reactive oxygen species (ROS) are inevitable by-products of metabolism [39]. However, when plants are exposed to stress, production of ROS is often induced in excess. When accumulated in excess, ROS may oxidize lipids, nucleic acids, and proteins destructively, causing developmental deficiency and ultimately resulting in cell death [40,41]. ROS can be removed by both enzymatic antioxidant defense systems in plants. The key antioxidant enzymes in the enzymatic route are POD, SOD, and CAT [42]. There have been many excellent reports that application of exogenous BRs can improve the POD, SOD, and CAT activities of plants facing abiotic stress, including *Solanum lycopersicum* under polychlorinated biphenyls stress, *Lycopersicon esculentum* under low-temperature stress, *Oryza sativa* under salinity and iron toxicity stress, *Vigna unguiculata* under water deficit stress, and *Ficus concinna* var. *subsessilis* under high-temperature stress [6,43,44,45,46,47]. There are also many excellent studies on the effect of ZnO nanoparticles on the activity of antioxidant enzymes in plants under stress. Their results show that ZnO NPs can increase the activity of antioxidant enzymes in plants under abiotic stress, such as *Carthamus tinctorius* L. under salt stress, *Cucumis sativus* L. under drought stress, *Linum usitatissimum* L. and *Triticum aestivum* under Cd-exposed stress, and *Leucaena leucocephala* (Lam.) under Cd and Pb exposure stress [11,14,48,49,50]. The present study showed that the antioxidant enzyme activities of seeds during the developmental process of *S. tonkinensis* were increased by EBL and ZnO NPs treatments, which may be beneficial for seed development and quality improvement. Moreover, the effects of EBL and ZnO NPs can be superimposed at the concentration used in this study. However, a limitation of this study is that only one concentration was used for both EBL and ZnO NPs; hence, future experiments using numerous concentrations are required.

### 4.2. Key Genes in Hormone Signaling Pathways

In addition to regulating plant growth and development under normal conditions, plant hormones also respond to various environmental stresses to regulate plant growth adaptability [51]. In our study, 64 key genes involved in plant hormone signaling were identified in the first two periods, located in the pathways of several different hormones, which implied that EBL and ZnO NPs treatment induced a complex hormonal crosstalk response in seeds. When plants are under stress, various plant hormones do not act alone, and complex crosstalk often occurs among them to cope with the changing environment [24]. In this study, under EBL and ZnO NPs treatment, many hormone signaling pathways, such as auxin, abscisic acid, jasmonic acid, brassinosteroid, and ethylene, were involved in the improvement in stress resistance of *S. tonkinensis* seeds. We analyzed the unique hormone-signaling-related genes induced by EBL and ZnO NPs treatment either alone or in combination (Supplementary Table S4). 

JZA is a key negative regulator of JA signaling in plant resistance to stress [52]. On 70 DAF, spraying EBL probably promoted JA signaling by downregulating expression of JAZ (TRINITY_DN22985_c1_g1), leading to an improvement in seed resistance. Iaa-amido synthetase belongs to the GH3 family and maintains IAA homeostasis by regulating coupling of IAA with amino acids [53]. MYC2 is a vital transcription factor in the JA signaling pathway and regulates plant response to abiotic stress [54]. As shown in Supplementary Table S4, on 70 DAF, upregulation of expression of Iaa-amido synthetase (TRINITY_DN109_c1_g3) and MYC2 (TRINITY_DN57526_c1_g3) was observed under zinc oxide treatment. These two genes may have contributed to the improvement in seed stress resistance of *S. tonkinensis* by ZnO NPs treatment. When the BR concentration is low, BKI1 exerts its effects by binding to the C-terminal tail of BRI1 and is a negative regulator of BR signaling [55]. On 110 DAF, the BR signal may be enhanced under ZnO NPs treatment because expression of BKI1 (TRINITY_DN1010_c0_g4) is downregulated. BSK is a crucial signal kinase in the BR signaling pathway, which could be significantly expressed in response to abiotic stress [56]. On 110 DAF, under EBL and ZnO NPs treatments alone or together, expression of BSK (TRINITY_DN97221_c0_g1) was upregulated. This means that BR is involved in hormone crosstalk by the EBL and ZnO NPs treatments alone or together. Aux/IAA is a transcriptional repressor that is polyubiquitinated and degraded when IAA concentrations rise [57]. On 110 DAF, expression of Aux/IAA was downregulated under both EBL (the Aux/IAA here are TRINITY_DN554_c0_g2 and TRINITY_DN82743_c0_g1) and ZnO NPs (the Aux/IAA here are TRINITY_DN4443_c0_g4 and TRINITY_DN554_c0_g5) treatments. This suggests that IAA signaling is induced under both EBL and ZnO NPs treatments, leading to an increase in IAA concentration. We also found that expression of ABF (TRINITY_DN1874_c0_g1) and PYL (TRINITY_DN21646_c0_g1), the positive regulators in the ABA signaling pathway, was upregulated under ZnO NPs treatment on 110 DAF. However, on 70 DAF, ABF (TRINITY_DN38284_c0_g1) expression was downregulated under ZnO NPs treatment. It was suggested that the mechanism of improving the stress resistance of ZnO NPs varies with the seed development stage. In fact, not only ABA signals, as shown above, but also EBL and ZnO NPs may activate different hormone signals at different stages of seed development to adapt to possibly different abiotic stresses, except for some positive genes related to the ABA signaling pathway that were upregulated at all stages and under all treatments. In addition to the above genes, SAUR and TGA showed some differences in expression under certain treatments. However, because SAUR is a positive/negative regulator in the IAA signaling pathway [58] and TGA is a positive or negative regulator in the SA signaling pathway [59], the functions of the SAUR and TGA genes screened in this study cannot be determined at present.

Among many plant hormones, the abscisic acid (ABA) signaling pathway plays a crucial role in plant response and adaptation to various environmental stresses [60]. ABA signaling is first sensed by PYL, and then ABA-bound PYL induces PP2C (EC:3.1.3.16) to release SNRK2 (EC:2.7.11.1), which then phosphorylates downstream transcription factors, such as ABF, and finally regulates expression of related genes [61]. Furthermore, ABA also critically mediates hormonal crosstalk at the transcriptional level in response to abiotic stress [62]. In our study, on 70 DAF, under treatment of both EBL and ZnO NPs together, upregulation of positive regulators PYL (TRINITY_DN21646_c0_g1; TRINITY_DN21646_c0_g2) and SNRK2 (TRINITY_DN1580_c0_g2) and downregulation of negative regulator PP2C (TRINITY_DN450_c0_g3) were found, while no changes in PYL and PP2C expression were found under other treatments. Additionally, on 110 DAF under treatment of both EBL and ZnO NPs together, there is also upregulation of ABF (TRINITY_DN1842_c0_g1) expression. This may highlight the role of ABA signaling in the superior effect treating both EBL and ZnO NPs together over either EBL or ZnO NPs alone. Moreover, on 70 and 110 DAF, genes in the ABA signaling pathway were found in the intersections of hormone signaling genes induced by all the treatments. In particular, on 70 DAF, the intersections of genes induced by all the treatments were only SNRK2, a protein phosphokinase at the end of ABA signaling. The findings above indicate the special significance of ABA signaling for seed resistance of *S. tonkinensis* under stress.

### 4.3. Late Stage of Seed Development

In this study, the module representing late seed development (Blue) was far more enriched in the ribosomal pathway than in other pathways (Figure 6C). The possible reason is that seeds focus on accumulating nutrients and synthesizing storage proteins and become dry in preparation for future germination at later stages of seed development [63]. In particular, in addition to storing protein, the seed also induces the production of a hydrophilic protein, LEA protein, at this time [64]. The LEA protein acts as a hydration buffer to protect cellular structures from water loss by sequestering ions, either by directly protecting other proteins or membranes or by denaturing unfolded proteins [65]. Additionally, being induced by reduced water during later stages of normal seed development, LEA is also induced by cellular water deficiency resulting from abiotic stresses, such as drought, salinity, osmotic pressure, cold and freezing temperatures, etc. [66]. In this study, the effect of ZnO NPs and ZnO NPs and EBL together on improving the antioxidant enzyme activity of seeds of *S. tonkinensis* was better than that of EBL alone, and this was no exception in the later stage of seed development. Therefore, it is possible that, on day 130 after anthesis, the seeds were still under stress in EBL or ZnO NPs treatment alone, so LEA was synthesized by ribosomes in large quantities. However, under the combination treatment of the two, the antioxidant enzyme activity of the seeds was further improved, and the concentration of reactive oxygen species was balanced within the normal range so that the seeds could concentrate on synthesis of other storage proteins and nutrients. Therefore, we identified 28 LEA-related genes from the total number of genes (Supplementary Table S5). As can be seen from Supplementary Table S5, with the maturation and senescence of seeds, expression of LEA-related genes increased, especially in the late-ripening stage. Additionally, at the late stage of maturation, expression of LEA-related genes was lowest under the combined treatment of both EBL and ZnO NPs. Therefore, the results supported the above hypothesis. Alternatively, LEA synthesis in *S. tonkinensis* seeds was brought on by both abiotic stress and seed maturation.

## 5. Conclusions

During *S. tonkinensis* seed development, foliar spraying of EBL and/or ZnO NPs could certainly boost seed stress resistance, as evidenced by an increase in the activities of antioxidant enzymes. The positive effects of both regulators could be stacked. The effects of EBL and ZnO NPs on 70 and 100 DAF were mostly due to complicated plant hormone crosstalk signaling, which involves IAA, JA, BR, and ABA signaling. Furthermore, ABA played an influential role in the hormone crosstalk. On 130 DAF, however, the increase in seed stress resistance brought about by EBL and ZnO NPs was linked to seed protein synthesis, particularly LEA production. Hence, this study may be helpful in further research in improving the cultivation techniques of *S. tonkinensis* and other woody biodiesel species to increase the economic value that could be obtained. Therefore, this work can be useful for future research into enhancing the cultivation methods of *S. tonkinensis* and other woody biodiesel species to boost the potential economic value.

## Figures and Tables

**Table 1:** The specific design of spraying treatments.

Treatments	The Specific Composition
CK	clean water
T_1_	5 mL/L EBL
T_2_	50 mL/L ZnO NPs
T_3_	5 mL/L EBL + 50 mL/L ZnO NPs

**Table 2:** Unigenes statistics identified for seeds of *S. tonkinensis*.

Type	Unigene	Transcript
total number	213,566	329,559
total base	238,692,947	405,460,296
largest length (bp)	16,047	16,047
smallest length (bp)	201	201
average length (bp)	1117.65	1230.31
N50 length (bp)	1586	1807
GC percent (%)	47.46	46.00

## Data Availability

Online repositories contain the datasets used in this investigation. The following list includes the name(s) of the repository(s) and the accession number(s): BioProject database at the National Center for Biotechnology Information (NCBI), accession number PRJNA886484.

## Supplementary Materials

The following supporting information can be downloaded at: https://www.mdpi.com/2073-4425/13/11/2170/s1, Table S1: The letter that represents each treatment (The letters with numbers 1,2, and 3 are used to represent the three biological replicates of the treatment); Table S2: Correlations among three biological replicates per treatment; Table S3: The gene expression variation of unigenes in *S. tonkinensis* seeds between treatment samplings and control group. These unigenes need to meet the threshold that *p* < 0.05 and | log2FC | ≥ 1; Table S4: Induction genes involved in plant hormone signaling for a specific treatment; Table S5: 28 LEA-related genes from the total number of genes.
